# Inference of Vohradský's Models of Genetic Networks by Solving Two-Dimensional Function Optimization Problems

**DOI:** 10.1371/journal.pone.0083308

**Published:** 2013-12-30

**Authors:** Shuhei Kimura, Masanao Sato, Mariko Okada-Hatakeyama

**Affiliations:** 1 Graduate School of Engineering, Tottori University, Tottori, Japan; 2 National Institute for Basic Biology, Okazaki Institute for Integrative Bioscience, National Institute for Natural Sciences, Okazaki, Japan; 3 RIKEN Center for Integrative Medical Sciences, Yokohama, Japan; Leibniz-Institute for Farm Animal Biology (FBN), Germany

## Abstract

The inference of a genetic network is a problem in which mutual interactions among genes are inferred from time-series of gene expression levels. While a number of models have been proposed to describe genetic networks, this study focuses on a mathematical model proposed by Vohradský. Because of its advantageous features, several researchers have proposed the inference methods based on Vohradský's model. When trying to analyze large-scale networks consisting of dozens of genes, however, these methods must solve high-dimensional non-linear function optimization problems. In order to resolve the difficulty of estimating the parameters of the Vohradský's model, this study proposes a new method that defines the problem as several two-dimensional function optimization problems. Through numerical experiments on artificial genetic network inference problems, we showed that, although the computation time of the proposed method is not the shortest, the method has the ability to estimate parameters of Vohradský's models more effectively with sufficiently short computation times. This study then applied the proposed method to an actual inference problem of the bacterial SOS DNA repair system, and succeeded in finding several reasonable regulations.

## Introduction

With the rapid advancement of technologies such as RNA-seq using next generation sequencers, it has become possible to measure the expression levels of thousands of genes. These data implicitly contain enormous amounts of information on biological systems. In order to exploit these high-throughput technologies, we must develop a way of extracting hidden information from the observed data. The inference of genetic networks is considered a promising approach for extracting useful information from these data. In the genetic network inference, the information is extracted by inferring mutual interactions among genes from the time-series of the gene expression levels. The inferred model of the genetic network is conceived of as an ideal tool to help biologists generate hypotheses and facilitate the design of their experiments. Many researchers have thus taken an interest in the inference of genetic networks, and the development of this methodology has become a major topic in the field of bioinformatics and systems biology.

Numerous models for describing genetic networks have been proposed, and numerous algorithms based on individual models have been developed for the inference of genetic networks [1]–[14]. Among these models, this study focuses especially on sets of differential equations, as they can capture the dynamic behavior of gene expression. When we use the set of differential equations to describe a genetic network, its inference is generally defined as a problem of estimating the model parameters that produce time-series data consistent with the observed gene expression levels.

A linear model is one of the best-studied models based on a set of differential equations. Several inference methods based on the linear model have therefore been proposed [13], [15]. The computation times of these methods are reportedly very short. As the linear model requires that the system is operating near a steady state, however, it is unsuitable for analyzing the time-series of gene expression levels [13]. An S-system model is another well-studied model based on a set of differential equations [16], [17]. As several fundamental properties of biochemical systems are inherent in this model, a number of inference methods based on it have been proposed [18]–[28]. However, the number of parameters in the S-system model is larger. The number of the parameters in the linear model is 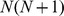, where 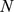 is the number of genes contained in the target network. On the other hand, the number of the parameters in the S-system model is 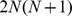. To obtain reasonable results, therefore, we should give the inference methods based on the S-system model a larger amount of the gene expression data.

When trying to infer genetic networks, we should use a mathematical model that has the ability to approximate actual biochemical reactions. As it is generally difficult to measure a sufficient amount of gene expression data, moreover, the model should contain a fewer number of the parameters. Vohradský proposed a model that is capable of representing the process of the gene expression [29]. The number of the parameters of the Vohradský's model, i.e., 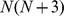, is comparable to that of the linear model. Because of its advantageous features, several researchers have proposed the inference methods based on this model [30]–[32]. However, these methods try to estimate all of the model parameters simultaneously. When inferring genetic networks consisting of many genes, therefore, they must solve high-dimensional non-linear function optimization problems. In order to overcome this high-dimensionality in the canonical methods, this study proposes a new approach that defines the estimation of the model parameters as two-dimensional function optimization problems. Although the defined problems are still non-linear, their low-dimensionality enhances the probability of obtaining reasonable results. Finally, we confirm the effectiveness of the proposed method by applying it to artificial and actual genetic network inference problems.

## Methods

### Vohradský's model

This study uses a mathematical model proposed by Vohradský [29] to describe genetic networks. The Vohradský's model is a set of differential equations of the form 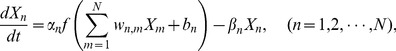
(1)where 
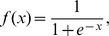
and 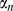 (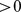), 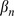 (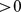), 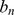 and 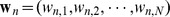 (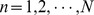) are model parameters. In the genetic network inference, 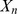 is the expression level of the 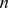-th gene and 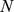 is the number of genes contained in the target network. When we use the Vohradský's model to describe genetic networks, our purpose is to estimate all of the model parameters that produce time-series data consistent with the observed gene expression levels.

The discrete form of the model (1) is equivalent to a recurrent neural network. We can thus use learning algorithms for recurrent neural networks, such as a back-propagation through time [33], in order to estimate the parameters of this model [29]. The canonical inference methods based on the Vohradský's model [30]–[32] have been designed on the basis of the back-propagation through time. In contrast to these methods, on the other hand, the proposed method estimates the parameters by solving simultaneous equations, as described below.

### Parameter estimation

The proposed method divides the inference problem of the Vohradský's model of a genetic network consisting of 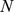 genes into 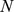 subproblems, each of which corresponds to each gene. By solving the 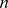-th subproblem, our method estimates the parameters corresponding to the 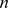-th gene, i.e., 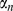, 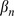, 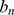 and 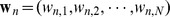. This section will describe the method to solve the 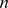-th subproblem.

### Concept

In the 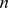-th subproblem corresponding to the 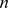-th gene, the proposed method estimates the model parameters, 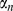, 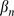, 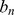 and 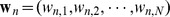, by solving the following simultaneous equations. 
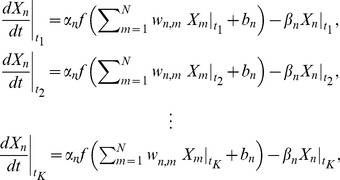
(2)where 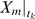 is the expression level of the 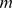-th gene at time 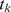, 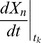 is the time derivative of the expression level of the 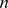-th gene at time 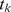, and 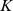 is the number of measurements. In the proposed approach, 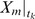
's are measured using technologies such as RNA-seq, and 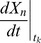
's are estimated directly from the observed time-series of the gene expression levels using a smoothing technique such as spline interpolation [34], local linear regression [35], neural networks [28], or a modified Whittaker's smoother [36]. Based on an idea similar to the method proposed here, several genetic network inference methods have already been proposed [8], [13], [23], [28], [37].

It is not always easy to solve the simultaneous equations (2), since they are non-linear. In order to resolve the difficulty in solving these equations, this study uses a feature arisen from the transformation of them. By rearranging the 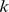-th member of the equations (2), we have 
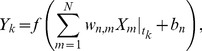
(3)where 
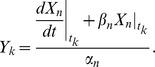



By applying 
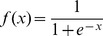
 to the equation (3), then, we obtain 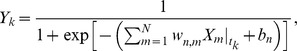


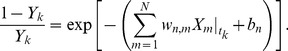
(4)


By taking the logarithms of both sides of the equation above, we finally have 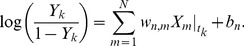
(5)


Note that, although the transformed equation (5) is non-linear with respect to the parameters 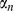 and 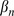, it is linear with respect to the parameters 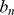 and 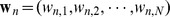. This fact suggests that, when the parameters 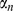 and 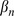 are given, the other parameters 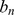 and 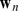 are easily estimated. The proposed method uses this feature for solving the simultaneous equations (2), as described below.

### Solving the simultaneous equations

As mentioned just before, we can easily estimate the parameters 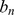 and 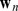, when the parameters 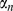 and 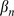 are given. In this study, the set of algebraic equations (2) is thus solved by estimating the parameters 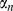 and 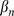. As this study uses a least-squares method to solve the simultaneous equations, the estimation of the parameters 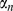 and 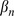 is defined as a problem of minimizing the following two-dimensional function. 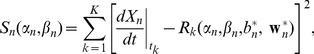
(6)where 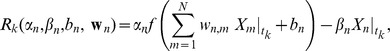
and 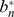 and 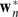 are the optimal values for 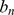 and 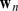, respectively, under given 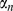 and 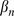. In the next section, we will describe a method for obtaining 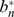 and 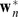.

Any function optimization algorithm can be used to minimize the objective function (6). When we used the local search for optimizing this function, however, several local optima were found. As this optimization problem seemed to be multimodal, this study uses an evolutionary algorithm, REX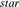/JGG (see Supporting Information) [38], to solve it. Because the parameters 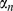 and 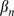 are positive, this study searches for them in a logarithmic space.

### Estimation of 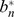 and 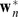


**Figure 1:**
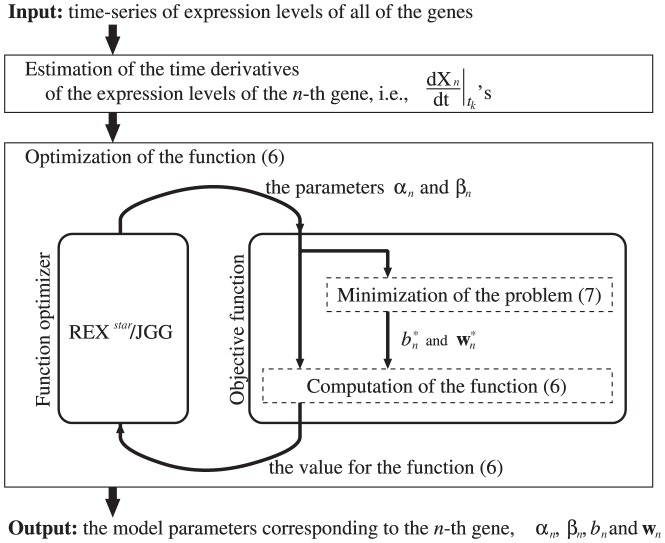
A framework of the algorithm for solving the 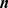-th subproblem. Note that the proposed method divides the inference of the Vohradský's model of a genetic network consisting of 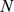 genes into 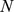 subproblems. By solving the 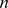-th subproblem, we have the model parameters corresponding to the 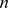-th gene, i.e., 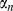, 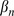, 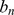 and 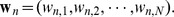

In order to compute a value for the objective function (6), we must provide values for 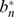 and 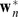. In the proposed method, they serve as the solution of a set of the transformed equations (5) under given 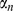 and 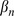. Note that, when the parameters 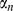 and 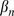 are given, these equations are linear with respect to the unknown parameters, i.e., 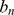 and 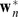. We can thus easily obtain 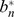 and 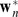. The proposed method estimates these parameters by optimizing the following constrained function minimization problem. 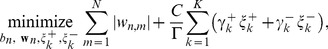
(7)subject to 
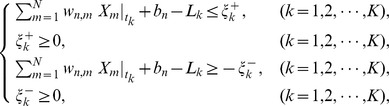
where 
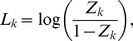


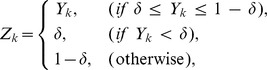


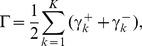


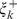
 and 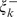 are slack variables, and 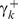, 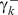, 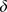 and 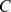 are constant parameters. In this problem, we treat the parameters 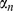 and 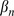 as constants. Note that, whenever trying to compute a value for the objective function (6), we must always solve the problem (7) (see Figure 1).

When the 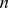-th gene is not regulated by the 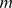-th gene, the parameter corresponding to this regulation, i.e., 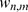, is zero in the Vohradský's model. Because genetic networks are known to be sparsely connected [39], most of 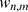
's should be zero. The first term of the objective function of the problem (7), i.e., 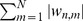, introduces this a priori knowledge into our parameter estimation. The second term of the objective function is, on the other hand, a sum of the differences between the left and right hand sides of the equations (5).

Note that, only when the condition 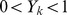 is satisfied, we can transform the equation (2) into the equation (5). However, the observed gene expression data are generally polluted by noise. Even when the optimum values are set for 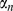 and 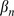, some 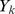
's might not satisfy the condition above. This study thus introduces a threshold parameter 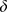, and sets its value to 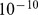. On the other hand, we should note that, when 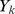 approaches 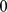 or 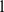, the term 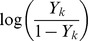 contained in the equation (5) approaches 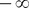 or 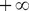, respectively. When 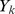 is approximately equal to 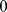 or 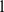, therefore, the transformation of the equation (2) into the equation (5) would amplify the noise contained in the measurement data. It is thus inadvisable to rely too much on the equations transformed under this condition. In order to introduce this notion into our estimation, this study sets the constant parameters 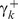 and 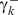 to 
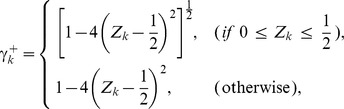


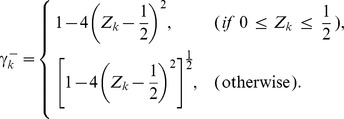



We can transform the optimization problem (7) to a linear programming problem. Thus, the proposed method easily solves this problem by using an interior point method [40].

### Remarks

As mentioned before, this study proposes to define the estimation of the model parameters corresponding to the 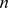-th gene as a problem of solving the simultaneous equations (2). The proposed method effectively solves them by minimizing the two-dimensional function optimization problem (6). We can however solve the simultaneous equations simply by using a least-squares method. In this case, for example, the parameters corresponding to the 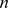-th gene are estimated by minimizing 
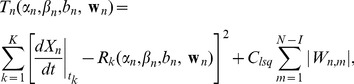
(8)where 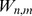
's are given by rearranging 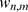
's in descending order of their absolute values, i.e., 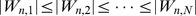. 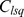 is a constant parameter, and 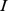 is a maximum indegree. The maximum indegree determines the maximum number of genes that affect the 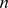-th gene directly. The second term of the objective function (8) is a penalty term that introduces the sparseness into the inferred network. Similar terms have been used in several genetic network inference methods [21], [22], [24].

As mentioned before, the existing inference methods based on the Vohradský's model try to estimate all of the model parameters simultaneously [30]–[32]. It is therefore difficult for them to analyze genetic networks with many genes because of the high-dimensionality in the parameter estimation. For the computational simplicity, moreover, they limit the model to being 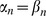. This study thus compared the proposed method chiefly with a method that minimizes the objective function (8). In this study, we refer to the method of optimizing this function as the least-squares approach. As same as the proposed method, the least-squares approach also uses REX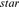/JGG [38] as a function optimizer. The following recommended values were used for the parameters of REX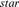/JGG applied in the least-squares approach; the population size 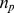 is 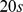, the number of children generated per selection 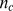 is 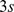, and the step-size parameter 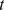 is 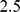, where 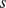 is the dimension of the search space. Each run was continued until the best objective value did not improved over 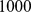 generations.

## Results and Discussion

This section shows that the proposed method has an ability to estimate parameters of Vohradský's models more effectively.

### Inference of a small-scale network

This experiment confirms that the proposed method is capable of estimating reasonable values for the parameters of the Vohradský's model.

**Experimental setup:** We used the Vohradský's model with 4 genes (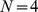), that was introduced by the reference [32], as a target network. The model parameters of this system are given in Table 1. Note that, as this network consists of 4 genes, the proposed method solves 4 individual two-dimensional function optimization problems to estimate all of the model parameters.

**Table 1:** The model parameters for the small-scale target network.

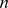	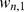	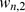	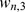	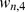	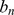	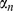	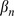
1	20.0	−20.0	0.0	0.0	0.0	0.1	0.1
2	15.0	−10.0	0.0	0.0	−5.0	0.2	0.2
3	0.0	−8.0	12.0	0.0	0.0	0.2	0.2
4	0.0	0.0	8.0	−12.0	0.0	0.2	0.2

The observed gene expression patterns, 3 sets of time-series data, each covering 4 genes, were computed from the differential equations (1) on the target model. The sets began from initial values randomly generated in 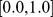, and 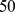 sampling points for the time-series data were assigned to each gene in each set. The number of observations 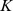 is therefore 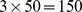. For a practical application, these sets would be obtained by actual biological experiments under different experimental conditions. This experiment simulated no measurement noise in the computed data. The time derivatives of the gene expression levels were thus directly computed from the target model. In this study, we estimated the parameters of the target model only from the gene expression levels and their derivatives.

We performed 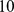 trials, each with different sets of gene expression data. We considered the model parameters to be successfully estimated only when the value of the objective function (6) dropped to less than 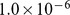. As the parameters 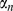 and 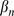 of our objective function (6) are both positive, this study searched for them in the logarithmic space. Their search area was set to 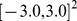. Based on the preliminary experiments, we set the constant parameter 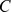 contained in the constrained function minimization problem (7) to 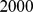. This study used the following recommended values for the parameters of the optimization algorithm, REX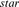/JGG (see Supporting Information) [38]: the population size 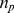 is 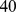, the number of children generated per selection 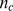 is 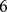, and the step-size parameter 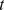 is 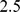. Each run of REX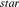/JGG was continued until the maximum number of the generation alternation reached 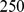. All of the computation were carried out on personal computers using Linux (Fedora release 12). The program was written in C++, and the compiler was gcc 4.4.2.

**Results:** The proposed method succeeded in estimating the parameter values with precision in 7 trials. Even in the rest of the trials, most of the parameters were correctly estimated. Table 2 shows a sample of the model parameters estimated in one of the failed trials. As mentioned before, the proposed method divided the parameter estimation problem of the target network here into 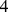 subproblems, each of which is defined as a two-dimensional function optimization problem. In this experiment, our method therefore solved 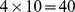 subproblems and failed to find the optimum solutions for only 3 of these 40 subproblems. While the averaged objective value (6) of the 3 failed subproblems was 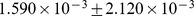, that of the other subproblems was 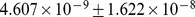. In order to estimate all of the model parameters for this network, our method took about 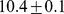 minutes on a single-CPU personal computer (Pentium IV 2.8 GHz).

**Table 2:** A sample of the parameters erroneously estimated by the proposed method in the experiment using the small-scale network.

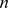	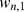	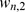	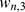	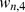	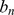	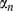	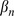
1	2.745	−0.662	0.090	−0.189	−1.667	0.730	0.457
2	15.002	−10.002	0.000	0.000	−5.000	0.200	0.200
3	0.043	−8.095	11.958	0.072	0.017	0.200	0.200
4	0.000	0.000	8.000	−12.000	0.000	0.200	0.200

Note that the proposed method succeeded in estimating the parameter values with precision in 7 of the 10 trials.

The discrete form of the Vohradský's model can be viewed as a recurrent neural network. The existing inference methods [30]–[32] have therefore designed on the basis of the learning algorithm for the recurrent neural network, i.e., the back-propagation through time [33]. For the computational simplicity, however, these methods limit the search space to 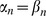. For making a fair comparison, thus, this study constructed two inference methods based on the back-propagation through time, i.e., BPTTLS and BPTTGA, that do not limit the search space. As function optimization algorithms, BPTTLS and BPTTGA used a local search, i.e., the conjugate gradient method [34], and an evolutionary algorithm, i.e., REX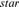/JGG [38], respectively. In Supporting Information, readers can find more detailed information on these inference methods. We then compared the proposed method with BPTTLS and BPTTGA. The computational costs of these methods were both lower in the small-scale problem described here. They were however unable to estimate the model parameters with precision. In order to estimate all of the model parameters, BPTTLS and BPTTGA required about 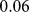 seconds and 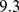 minutes, respectively, on the single-CPU personal computer (Pentium IV 2.8GHz). The averaged objective values of BPTTLS and BPTTGA were 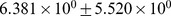 and 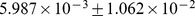, respectively. The objective values of BPTTLS were much worse than those of BPTTGA. Note here that, when we set the model parameters to their optimal values, its objective value is better than those of BPTTGA. These facts indicate that the objective function defined by the back-propagation through time has a lot of local optima. A typical sample of the model parameters estimated by BPTTGA was shown in Table 3. Although the existing inference methods [30]–[32] limit the search space, on the other hand, they were reportedly still unable to estimate the model parameters with precision.

**Table 3:** A typical sample of the parameters estimated by BPTTGA.

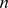	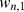	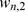	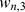	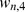	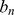	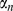	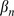
1	20.559	−19.907	0.396	−0.309	0.079	0.098	0.100
2	13.422	−8.879	−0.633	1.296	−4.593	0.190	0.189
3	−7.843	0.037	−2.923	−1.354	−0.728	3.000	0.000
4	−0.079	−0.016	7.516	−10.387	0.022	0.161	0.188

BPTTGA is the parameter estimation method of Vohradský's models, that is designed on the basis of the back-propagation through time [33]. As a function optimizer, BPTTGA used an evolutionary algorithm, REX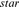/JGG [38]. Readers can find more detailed information on BPTTGA in Supporting Information.

### Inference in noisy environment

Next, we checked the performance of the proposed method in a real-world setting by conducting an experiment with noisy data.

**Experimental setup:** In the second experiment, we used the Vohradský's models consisting of 10, 20 and 30 genes (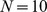, 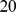 and 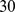) as target networks. As the inference ability of our method might depend on the structure of the target network, we generated the target networks of different structures by changing the model parameters. When trying to determine the model parameters corresponding to the 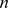-th gene, we randomly chose an integer 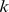 from a power-law distribution with a cutoff of 5. Then, 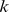 genes were randomly selected from all of the genes contained in the network. The weight parameters 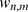
's corresponding to the regulations of the 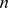-th gene from the selected genes were randomly chosen from 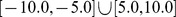, and the rest of the weight parameters were set to 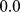. We also randomly selected the parameters 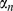 and 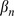 from 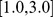. The parameter 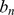 was set to 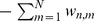. This study changed the network structure on every trial.

As the performance of the inference method might also depend on the amount of time-series data given, different numbers of time-series datasets were used for the experiments. The time-series datasets were obtained by solving the differential equations (1) on the target networks. The initial values of these sets were selected randomly from 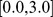. Each dataset consisted of the expression levels at 11 time points with 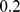 time intervals. The measurement noise was simulated by adding 10% Gaussian noise to the computed time-series data. To estimate the time derivatives of the gene expression levels from the given time-series datasets, this experiment used the local linear regression [35], an interpolation technique.

In order to check the performance of the proposed method, this study constructed and then solved 10 genetic network inference problems of each available size with each available number of time-series datasets. In this experiment, we set the constant parameter 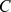 to 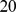. All of the other experimental conditions were the same as those used in the previous experiment.

**Results:** In the noisy environment, the proposed method was unable to estimate the parameter values with precision. In this experiment, therefore, we only checked whether or not our method infers the structures of the target networks correctly. Note that the Vohradský's model represents the positive and negative regulations from the 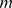-th gene to the 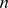-th gene as positive and negative values, respectively, of the weight parameter 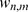. On the other hand, when the 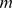-th gene has no influence on the 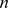-th gene, the value of the parameter 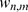 is zero. This study thus extracted the structures of the networks from the estimated model parameters according to the following rules: when 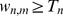 and 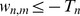, we conclude that the 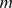-th gene positively and negatively, respectively, regulates the 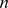-th gene, where 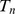 is a threshold; otherwise, we infer no regulation from the 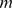-th gene to the 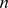-th gene. This study set the threshold 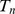 to 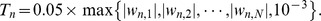



**Figure 2:**
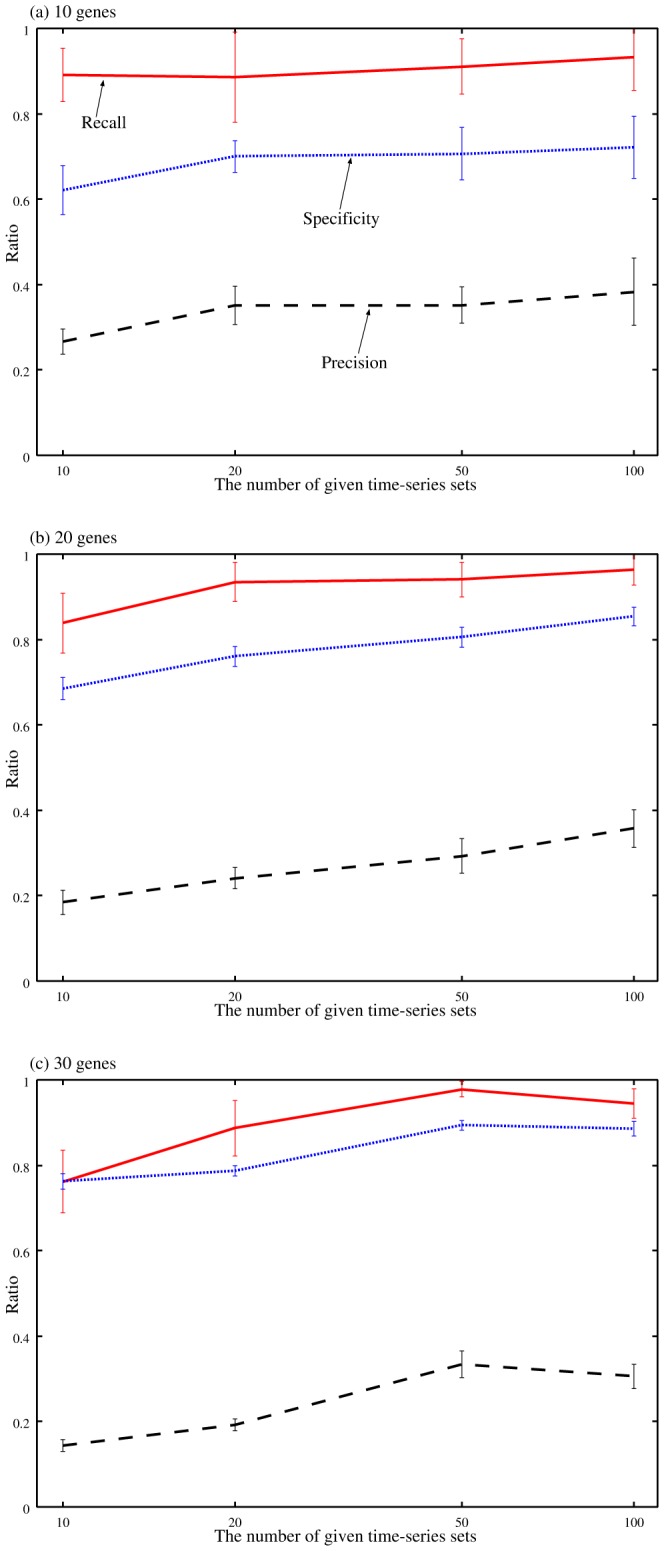
The performances of the proposed method on the experiments of genetic networks consisting of (a) 10 genes, (b) 20 genes, and (c) 30 genes, respectively. Solid, dotted and dashed lines represent the recall, the specificity and the precision, respectively.

**Figure 3:**
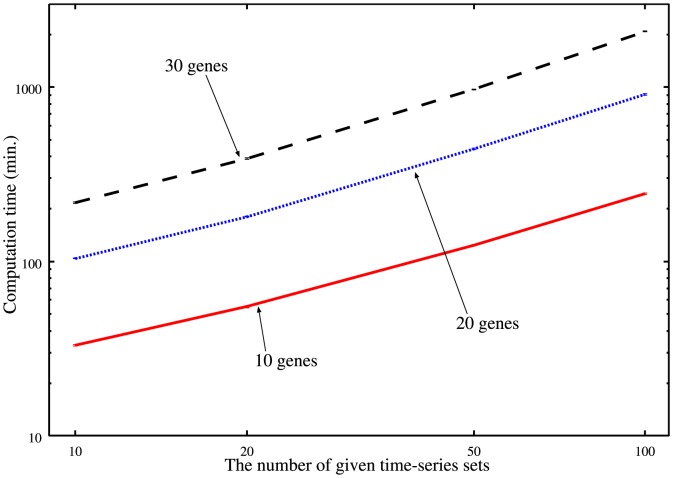
The computation times of the proposed method for noisy experiments. Solid, dotted and dashed lines represents the averaged computation times required for solving the inference problems for 10, 20 and 30 genes, respectively.

Figure 2 (a), (b) and (c) show the recalls, the precisions and the specificities of the proposed method on the experiments of solving the inference problems for different sizes with different amounts of gene expression data. The recall, the precision and the specificity are defined as 
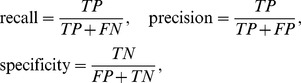



where 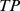, 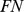, 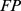 and 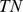 are the numbers of true-positive, false-negative, false-positive and false-negative regulations, respectively. The figures show that the performances of the proposed method improved with increasing the amount of the given data. A similar experiment has been performed to confirm the performance of the inference method based on the S-system model [23]. These results indicate that the proposed method has the ability to infer a more reasonable network even with a smaller amount of gene expression data. This advantageous feature is due to the smaller number of the parameters of the Vohradský's model. On the other hand, the computation time required by the proposed method was not always short. The computation times of our method on the single-CPU personal computer (Pentium IV 2.8 GHz) are shown in Figure 3.

**Figure 4:**
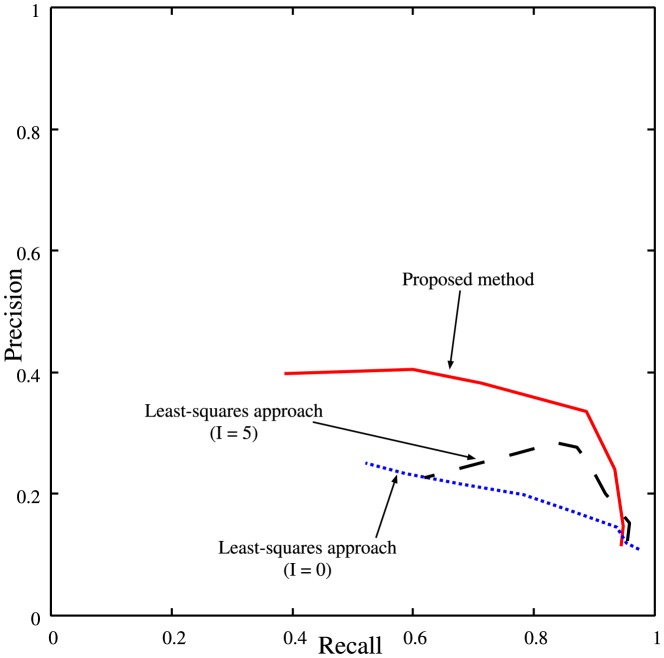
The precision versus the recall for the genetic network inference problems of 20 genes with 20 sets of time-series data. A solid line represents the performances of the proposed method. Dotted and dashed lines represent the performances of the least-squares approach with 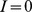 and 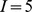, respectively.

The existing inference methods based on the Vohradský's model try to estimate all of the model parameters simultaneously [30]–[32]. Because of the high-dimensionality in their parameter estimation, it is difficult for them to analyze genetic networks consisting of dozens of genes. When we applied BPTTGA mentioned before to the inference problem of 20 genes with 20 sets of time-series data, therefore, its computation did not finish within 72 hours. Although the computational cost of BPTTLS was still low, on the other hand, its recalls, precisions and specificities were much worse. We thus compared the proposed method only with the least-squares approach described before. Figure 4 shows the precision-recall curves of the proposed method and the least-squares approach on the genetic network inference problems of 20 genes with 20 sets of noisy time-series data. The least-squares approach was performed under different hyper-parameter settings, i.e., 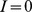 and 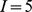. These curves were obtained by changing the hyper-parameter of our method, i.e., 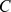, and that of the least-squares approach, i.e., 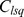. The figure indicates that our method outperforms the least-squares approach with respect to inference ability. However, the computation time of the proposed method was much longer. While the least-squares approach required 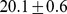 minutes on the single-CPU personal computer (Pentium IV 2.8 GHz) to infer each network, the proposed method required 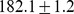 minutes on the same computer. In the future work, we must therefore develop a way to reduce the computational cost of the proposed method.

In the proposed method, the number of the inferred regulations depends on the value of the hyper-parameter 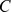. Figure 4 indicates, on the other hand, that the quality of the network inferred by the proposed method was quickly degraded with decreasing the number of the inferred regulations. When trying to analyze an actual genetic network, therefore, we should set the hyper-parameter 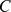 so that the inferred network contains a larger number of regulations.

### Analysis of actual data

We then checked the performance of the proposed method in an experiment using actual gene expression data.

**Experimental setup:** In this experiment, we analyzed the SOS DNA repair regulatory network in *E.coli* [41]. More than 30 genes are known to be involved in this system. This study however analyzed the expression data of six genes, i.e., *uvrD*, *lexA*, *umuD*, *recA*, *uvrA* and *polB*, which had been measured by Ronen and colleagues [42] (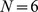). These data have often been used to confirm the performances of the inference methods [7]–[9], [18], [19], [23], [31], [32]. The original expression data contain four sets of time-series data. This study however used only two sets (the third and fourth sets), since those two had been measured under the same experimental conditions. Each set of time-series data consisted of 50 measurement values including the initial concentrations of zero. In the experiment, we removed the initial concentrations from both of the sets, as models based on a set of differential equations cannot produce different time-courses from the same initial conditions. The number of measurements 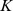 is thus 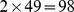. According to our previous work [9], we normalized the data corresponding to each gene against its maximum expression level. The normalized data were then smoothed by the local linear regression [35]. We assigned a value of 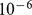 to expression levels with values of less than 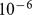, as the gene expression levels must not be negative.

In this experiment, we set the hyper-parameter 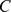 to 2000. All of the other experimental conditions were the same as those described before.

**Figure 5:**
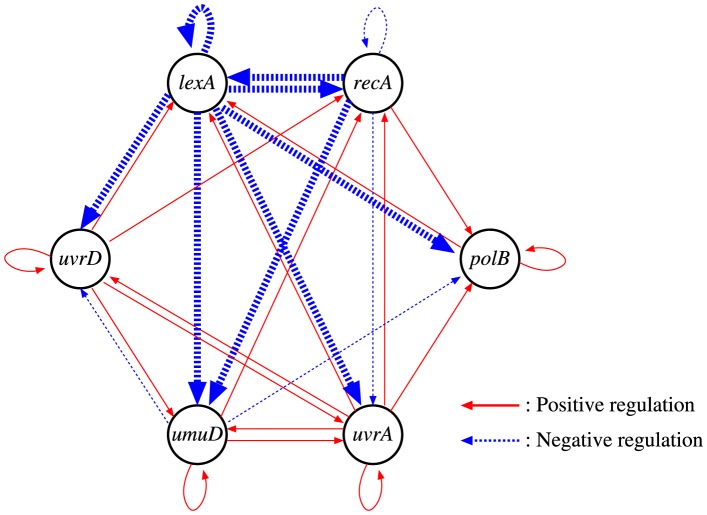
The network structure obtained for the SOS DNA repair system in *E.coli*. Bold lines represent biologically plausible regulations mentioned in the ‘Analysis of actual data’ section.

**Results:** Figure 5 shows the structure of the network inferred by the proposed method. Although we performed 10 trials in this experiment, all of the inferred networks had the same structure. A sample of the parameters estimated by the proposed method is listed in Table 4. As shown in the figure, our method removed few regulations from all of the candidate regulations. Most of the inferred regulations would therefore be false-positive. However, the negative regulations of all of the genes from *lexA* are reasonable, since LexA is known to repress the SOS genes. The negative regulation of *lexA* from *recA* also appears to be reasonable, as RecA senses the damage of DNA and mediates LexA autocleavage. Moreover, the regulation of *umuD* from *recA*, inferred by the proposed method, has been contained in a network now known [15].

**Table 4:** A sample of the parameters estimated by the proposed method in the experiment with actual gene expression data.

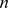	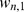	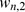	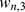	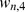	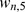	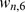	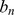	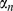	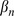
1(*uvrD*)	4.412	**−6.461**	**−**0.354	0.000	5.900	**−**0.028	**−**4.183	0.336	0.055
2 (*lexA*)	6.056	**−14.288**	0.000	**−7.567**	19.773	1.668	**−**4.769	0.163	0.050
3 (*umuD*)	3.291	**−11.751**	7.892	**−5.101**	9.328	0.475	**−**4.997	0.604	0.095
4 (*recA*)	4.173	**−14.738**	6.996	**−**3.642	11.510	0.000	**−**5.195	0.464	0.069
5 (*uvrA*)	5.230	**−20.042**	9.914	**−**7.364	19.515	0.180	**−**4.307	0.259	0.246
6 (*polB*)	**−**0.368	**−4.866**	**−**17.080	10.910	15.702	7.342	**−**2.536	0.094	0.110

The parameters written in boldface type correspond to biologically plausible regulations mentioned in the ‘Analysis of actual data’ section.

As mentioned above, our method seemed to find a number of false-positive regulations. In a future work, therefore, we should find a way to reduce these erroneous regulations.

### Experiments on a DREAM3 network

In the experiments described before, we focused on whether the proposed method has an ability to estimate parameters of the Vohradský's models more effectively. Therefore, our experiments have chiefly used the Vohradský's models as the target networks. In the experiments described here, on the other hand, we applied the proposed method to an inference problem whose target network is described as a set of differential equations of the form different from the Vohradský's model.

**Experimental setup:** The proposed method was applied to one of the artificial genetic network problems obtained from DREAM3 in silico challenges [43]. These problems have been often used to check the performances of genetic network inference methods. This study analyzed the third network, i.e., Yeast1, which consists of 100 genes.

46 sets of time-series data, that were obtained by solving a set of differential equations of the form different from the Vohradský's model, were given as the observed gene expression levels. The given data were polluted by noise. 21 sampling points for time-series data were assigned on each gene in each set. The number of observations 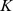 was therefore 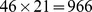.

In this experiment, we checked the performances of the proposed method by changing a value for the hyper-parameter 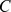 from 20 to 2000. All of the other experimental conditions were the same as those described before.

**Results:** The performances of the proposed method on the DREAM3 problem are shown in Table 5. The network inferred by the proposed method tells us whether the regulation of the 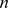-th gene from the 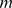-th gene is positive or negative. As the correct network given by the DREAM3 does not have the information about the types of the regulations, however, we omitted to check the types of the regulations. Compared with the champion algorithm of the DREAM3 challenges [44], the performances of our method were worse. Note however that this study confirmed the effectiveness of the proposed method through the experiments on the actual genetic network inference problem. Thus, the experimental results shown here do not always prove the inability of the proposed method in analyzing actual gene expression data. The Vohradský's model would be unsuitable to capture the features of the DREAM3 network. One of the reasons of the unsuitability would be that, while the model used in the DREAM3 problem considers the effect of the intrinsic noise [45], the Vohradský's model does not consider it. The intrinsic noise is unavoidable in biological processes, and the analysis of it would be important to understand biological systems. However, the current technologies generally measure the gene expression levels averaged over a lot of cells. As we think that the averaged gene expression levels weaken the effect of the intrinsic noise, the method proposed in this study infers genetic networks without considering it.

**Table 5:** The performances of the proposed method on the third problem of the DREAM3 in silico challenges [43].

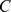	FP	FN	TP	TN	recall	precision	specisificy
20	15	153	13	9719	0.078	0.464	0.998
50	78	131	35	9656	0.211	0.310	0.992
100	312	112	54	9422	0.325	0.148	0.968
150	632	95	71	9102	0.428	0.101	0.935
200	876	91	75	8858	0.452	0.079	0.910
500	1948	84	82	7786	0.494	0.040	0.800
2000	4009	69	97	5725	0.584	0.024	0.588

The performances were checked by changing the hyper-parameter of the proposed method, 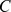. FP, FN, TP and TN are the numbers of false-positive, false-negative, true-positive, true-negative regulations, respectively.

## Conclusion

This study proposed a new method for the inference of Vohradský's models of genetic networks. The proposed method resolves the difficulty in the estimation of the model parameters by defining it as two-dimensional function optimization problems. The experimental results indicated that our method has an ability to estimate reasonable values for the parameters of the Vohradský's model. However, the computation time of the proposed method is not always short. In the future work, therefore, we must develop a technique to reduce its computational cost.

A variety of inference methods based on a variety of mathematical models have been proposed. However, we still do not know which method is the most suitable for the inference of genetic networks. In order to obtain a reliable network, therefore, it will be important to analyze the measurement data using multiple inference methods based on models different from each other. The inference method proposed in this study may be a promising choice for this purpose.

## Supporting Information

Text S1
**Detailed algorithms of REX** 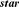 **/JGG and the least-squares approach.**
(PDF)

Text S2 **Time-series data used in the ‘Inference in noisy environment’ section (compressed by gzip).**
(GZ) Click here for additional data file.
