# Supplementary material for: Inference of Vohradský's Models of Genetic Networks by Solving Two-Dimensional Function Optimization Problems
Source: PLoS One. 2013 Dec 30;8(12):e83308. doi: 10.1371/journal.pone.0083308 (PMC3875442; doi:10.1371/journal.pone.0083308)
Supplement: Text S1 — Detailed algorithms of REX /JGG and the least-squares approach. (PDF) [file pone.0083308.s001.pdf]

Supporting Information: Inference of Vohradský's models of genetic networks by solving two-dimensional function optimization problems

Shuhei Kimura *et al.*
kimura@ike.tottori-u.ac.jp

1 REX^{star}/JGG

REX^{star}/JGG [1] is a real-coded genetic algorithm, a sort of evolutionary algorithm, that uses JGG as a generation alternation model and REX^{star} as a recombination operator. This section will describe each of the operators in greater detail.

1.1 JGG

JGG is a generation alternation model. The following is an algorithm of JGG. In the algorithm described below, a recombination operator uses m (≥ 2) parents to generate offsprings.

[Algorithm: MGG]

1. **Initialization**
   As an initial population, create n_p individuals. As REX^{star}/JGG is a real-coded genetic algorithm, these individuals are represented as s-dimensional real number vectors, where s is the dimension of the search space. Set $Generation = 0$.
2. **Selection for reproduction**
   Select m individuals without replacement from the population. The selected individuals, that are expressed here as $\mathbf{p}_1, \mathbf{p}_2, \cdots, \mathbf{p}_m$, are used as the parents for the recombination operator in the next step.
3. **Generation of offsprings**
   Generate n_c children by applying the recombination operator to the parents selected in the previous step. This study uses REX^{star} as the recombination operator.
4. **Selection for survival**
   Select the best m individuals from the family containing the m parents ($\mathbf{p}_1, \mathbf{p}_2, \cdots, \mathbf{p}_m$) and their children. Then, replace the m parents with the selected individuals. In the original JGG, the best m individauls are selected only from the children. As its optimization process seemed to be unstable, however, this study slightly modified its algorithm.
5. **Termination**
   Stop if the halting criteria are satisfied. Otherwise, $Generation \leftarrow Generation + 1$, and then return to the step 2.

1.2 REX^{star}

REX^{star} is a real-coded crossover operator. REX^{star} uses $s + 1$ parents, where s is the dimension of the search space, and generate n_c ($> s + 1$) children according to the following algorithm. In the following algorithm, the parents are represented as $\mathbf{p}_1, \mathbf{p}_2, \cdots, \mathbf{p}_{s+1}$.

[Algorithm: REX^{star}]

1. Generate reflection points, $\underline{\mathbf{p}_1}, \underline{\mathbf{p}_2}, \cdots, \underline{\mathbf{p}_{s+1}}$, of the parents, i.e.,

$$\underline{\mathbf{p}_i} = 2\mathbf{G} - \mathbf{p}_i, \tag{1}$$

where

$$\mathbf{G} = \frac{1}{s+1} \sum_{i=1}^{s+1} \mathbf{p}_i.$$

2. Compute the objective values of the $s+1$ reflection points generated in the previous step. In REX^{star}, these reflection points are treated as the children.

3. From the parents and their reflection points, select the best $s+1$ individuals, and then compute the center of the gravity of the selected individuals. This study represents it as $\mathbf{G}^b$.

4. Generate $n_c - s - 1$ children by applying the following equation $n_c - s - 1$ times. Note that the $s+1$ reflection points generated in the step 1 are treated as the children. The total number of the children generated is therefore n_c.

$$\mathbf{c} = \mathbf{G} + diag(\xi_1^t, \xi_2^t, \cdots, \xi_s^t)(\mathbf{G}^b - \mathbf{G}) + \sum_{i=1}^{s+1} \xi^i (\mathbf{p}_i - \mathbf{G}), \tag{2}$$

where ξ_i^t's and ξ^i's are random numbers drawn from uniform distributions $[0, t]$ and $\left[-\sqrt{\frac{3}{s+1}}, \sqrt{\frac{3}{s+1}}\right]$, respectively, where t is a hyper-parameter named a step-size parameter.

2 Back-propagation through time

The discrete form of the Vohradský's model can be viewed as a recurrent neural network. The existing inference methods [2, 5, 6] have thus designed on the basis of the learning algorithm for the recurrent neural network, i.e., the back-propagation through time [4]. In the back-propagation through time, all of the parameters of the Vohradský's model are estimated simultaneously by minimizing

$$S(\alpha, \beta, \mathbf{w}, \mathbf{b}) = \sum_{n=1}^{N} \sum_{k=2}^{K} \left(X_n|_{t_k} - X_n|_{t_k}^{cal} \right)^2, \tag{3}$$

where $\alpha = (\alpha_1, \alpha_2, \cdots, \alpha_N)$, $\beta = (\beta_1, \beta_2, \cdots, \beta_N)$, $\mathbf{w} = (w_{1,1}, w_{1,2}, \cdots, w_{N,N})$, and $\mathbf{b} = (b_1, b_2, \cdots, b_N)$ are the model parameters, N is the number of genes contained in the network, and K is the number of measurements. $X_n|_{t_k}$ and $X_n|_{t_k}^{cal}$ are the observed and the computed expression level of the n-th gene at time t_k, respectively. In the back-propagation through time, $X_n|_{t_k}^{cal}$ is computed from the discrete form of the Vohradský's model, i.e.,

$$X_n|_{t_k} = \alpha_n f \left(\sum_{m=1}^{N} w_{n,m} \, X_m|_{t_{k-1}} + b_n \right) \Delta t + (1 - \beta_n \Delta t) \, X_m|_{t_{k-1}}, \tag{4}$$

where $\Delta t = t_k - t_{k-1}$.

This study constructed two inference methods based on the back-propagation through time, i.e., BPTTLS and BPTTGA. BPTTLS and BPTTGA used a local search, i.e., the conjugate gradient method [3], and an evolutionary algorithm, i.e., REX^{star}/JGG [1], respectively, as function optimization algorithms. The following recommended values were used for the parameters of REX^{star}/JGG applied here; the population size n_p is $20s$, the number of children generated per selection n_c is $3s$, and the step-size parameter t is 2.5, where s is the dimension of the search space. Each run was continued until the best objective value did not improved over $2 \times n_p$ generations.

References

[1] S. Kobayashi, "The frontiers of real-coded genetic algorithms," *Transactions of the Japanese Society for Artificial Intelligence*, Vol. 24, pp. 147–162, 2009 (in Japanese).

[2] L. Palafox and H. Iba, "On the use of population based incrementasl learning to do reverse engineering on gene regulatory networks," *Proc. 2012 Congress on Evolutionary Computation*, pp. 1865–1872, 2012.

[3] W.H. Press, S.A. Teukolsky, W.T. Vetterling and B.P. Flannery, Numerical Recipes in C, 2nd Edition, Cambridge University Press, 1995.

[4] R.J. Williams and J. Peng, "An Efficient Gradient-based Algorithm for On-line Training of Recurrent Network Trajectories," *Neural Computation*, Vol. 2, pp. 490–501, 1990.

[5] R. Xu and G.K. Venayagamoorthy and D.C. Wunsch II, "Inference of gene regulatory networks with hybrid differential evolution and particle swarm optimization," *Neural Networks*, Vol. 20, pp. 917–927, 2007.

[6] R. Xu, D.C. Wunsch II and R.L. Frank, "Inference of genetic regulatory networks with recurrent neural network models using particle swarm optimization," *IEEE/ACM Transactions on Computational Biology and Bioinformatics*, Vol. 4, pp. 681–692, 2007.
