# Supplementary material for: Inference of Vohradský's Models of Genetic Networks by Solving Two-Dimensional Function Optimization Problems
Source: PLoS One. 2013 Dec 30;8(12):e83308. doi: 10.1371/journal.pone.0083308 (PMC3875442; doi:10.1371/journal.pone.0083308)
Supplement: Text S2 — Time-series data used in the ‘Inference in noisy environment’ section (compressed by gzip). (GZ) [file pone.0083308.s002.gz › time_series_data/readme.pdf]

Time-series data used in `Inference of Vohradsky's models of genetic networks by solving two-dimensional function optimization problems'

There are sets of time-series data used in the `Inference in noisy environment' section. `gene10', `gene20' and `gene30' directories contain time-series data of the networks consisting of 10, 20 and 30 genes, respectively. The `gene10', `gene20' and `gene30' directories have `10sets', `20sets', `50sets' and `100sets' directories containing 10, 20, 50 and 100 sets of time-series data, respectively.
Each directory contains 10 files of the form `tc.dat*' and 10 files of the form `params.dat*'. The `tc.dat*' file is time-series data, and the `params.dat*' file is the parameters of the Vohradsky's model of the target network. The time-series data contained in the `tc.dat(number)' file were generated from the Vohradsky's model whose parameters are given in the `params.dat(number)' file.
